# Sampled-data stabilization of delayed Boolean control networks with state inequality constraints

**DOI:** 10.1038/s41598-025-02737-x

**Published:** 2025-05-21

**Authors:** Xiangshan Kong, Enguo Gu, Xinyun Liu, Yalu Li, Guanpeng Wang

**Affiliations:** 1 School of Mathematics and Statistics, Weifang University, Weifang, China; 2 Basic Teaching Department, Weifang Engineering Vocational College, Qingzhou, China; 3 School of Mathematics and Statistics, Qingdao University, Qingdao, China

**Keywords:** Delayed Boolean control networks, State inequality constraints, Stabilization, Nonuniform sampled-data control, Algebraic state space representation, Gene regulation, Proteins

## Abstract

This paper studies the set stabilization of delayed Boolean control networks (DBCNs) with state inequality constraints via time-variant nonuniform sampled-data control. State inequality constraints are introduced into DBCNs. Firstly, the equivalent algebraic forms of DBCNs and the solution set of state inequality constraints are constructed via the algebraic state space representation approach, based on which an inequality constrained controllability matrix is constructed. Then, by the inequality constrained controllability matrix, new criteria are proposed for the nonuniform sampled-data inequality constrained reachability of DBCNs. Finally, a time-variant nonuniform sampled-data stabilizers are designed for DBCNs by utilizing the nonuniform sampled-data inequality constrained reachability. The effectiveness of the obtained results is verified through the cell apoptosis network.

## Introduction

Boolean networks (BNs) are a special kind of nonlinear systems with logical operations^1,2^. The model of BNs was established by Kauffman to study gene regulatory networks (GRNs)^3^. Furthermore, BNs with inputs are referred to Boolean control networks (BCNs), which is a correct method to describe the dynamics of GRNs^4^. For some basic theory and applications of BNs, please refer to^5,6^. However, due to the lack of mathematical methods for handling logical processes, it becomes very inconvenient to study the control issues of BNs^7^.

Recently, a new matrix product, called semi-tensor product of matrices, has provided great convenience for the study of BNs^8^. Based on the characteristics of matrix product, an algebraic state space representation (ASSR) framework has been constructed for the research of BNs. Therefore, researchers can use the ASSR to investigate BNs through the traditional control theory^9^. In the past twenty years, many excellent results have been achieved for BNs via the ASSR framework^10,11^. Observability and controllability for BNs were investigated in^12,13^. Stability and stabilization of BCNs were investigated in^14–16^.

Control design is always a core issue in control theory^17,18^. Recently, many control design methods have been introduced to address the control problems of BCNs through the ASSR^19^. Furthermore, how to design appropriate control techniques to reduce control costs is an important topic in modern control theory. As we all know, sampled-data control (SDC) is an effective technique, which reduces the update frequency of the controller and significantly reduces the computational burden^20,21^. In^22^, a self-triggered implementation of the proposed event-triggered sampling scheme was presented. Based on the ASSR framework, SDC method is introduced into the control of BCNs^19,23^, and some basic results are proposed for the sampled-data stabilization and controllability of BCNs^24^. There are two types of SDC: uniform SDC and nonuniform SDC (NSDC). Since the sampling length of NSDC is time-varying, it can more effectively utilize information resources than uniform SDC^25^, which also makes the controller design more challenging.

In GRNs, time delay is generally used to represent slow biochemical reactions^26^. In addition, external environmental factors, such as nutrient concentration and temperature may also lead to time delays in GRNs. For example, the coupled oscillatory biochemical network in cell cycle is simplified as the delayed BN^27^:

1 $$\begin{aligned} \left\{ \begin{array}{lll} C_{1}(t+1)=\lnot \big (C_{1}(t-2)\wedge C_{2}(t-1) \big ),\\ C_{2}(t+1)=\lnot \big (C_{1}(t-1)\wedge C_{2}(t-2) \big ),\end{array}\right. \end{aligned}$$

where $C_{1}(t)$ and $C_{2}(t)$ represent the state of two cells at time *t*, and time delays are caused by the delayed translocation between cells. In the past ten or more years, delayed BNs have attracted the research interest of many scholars. Delayed BNs were firstly investigated through the ASSR in^8^. Subsequently, delayed BCNs (DBCNs) were also well investigated in some works^12,28^. In^29^, fault detectability of asynchronous DBCNs with sampled-data control was investigated.

As is well known, constraints play a very important role in nonlinear systems^30^. In GRNs, it is necessary to impose constraints on certain gene states that may lead to diseases and treatment options that may lead to serious complications^31^. For example, in WNT5A gene networks, states of the WNT5A=1 are undesirable as they accelerate the opportunity for transfer^32^. State inequality constraints, as an important form of constraints, often appear in the control logic design of dynamic systems^33^. They are usually physical constraints of the system and have wide practical applications^34^. For example, in a semi-batch reactor, due to the volume constraint of the reactor, only a limited amount of substrate can be fed. Similarly, for safety reasons, one may not want the reactor to operate above a certain maximum temperature. In a feed batch bioreactor, there may be constraints on cell mass concentration (beyond which oxygen transfer is restricted) or substrate concentration (beyond which unexpected side reactions may occur). However, there are no relevant results on the investigation of DBCNs with state inequality constraints via NSDC.

In this paper, based on the ASSR framework, we analyze the inequality constrained set reachability and inequality constrained set stabilization of DBCNs with state inequality constraints via NSDC. The main contributions can be summarized in two aspects: (i) State inequality constraints are introduced into DBCNs, and an inequality constrained controllability matrix is constructed for studying the inequality constrained set stabilization of DBCNs. (ii) NSDC provides us with more control schemes to achieve control objectives and reduce control costs. The traditional control and uniform SDC can be considered as a special case of NSDC.

The remainder of this paper is organized as follows: Section “Problem formulation” presents the equivalent system of DBCNs with state inequality constraints through the ASSR framework. The inequality constrained controllability matrix and nonuniform sampled-data inequality constrained reachability are considered in section “Inequality constrained set reachability”. The time-variant state feedback inequality constrained set stabilization of DBCNs is studied in section “Inequality constrained set stabilization”. Section “Illustrative example” and section “Conclusions” provide an illustrative example and a brief summary, respectively.

*Notations:* $\mathbb {R}$, $\mathbb {Z}$, $\mathbb {N}$ and $\mathbb {Z_{+}}$ represent the set of real numbers, integers, nonnegative integers and positive integers, respectively. $\textbf{0}_{s}:=(\underbrace{0,\cdots ,0}_{s})^{\top }$. $\textbf{1}_{s}:=(\underbrace{1,\cdots ,1}_{s})^{\top }$. $I_s$ denotes the *s*-order identity matrix. $(A)_{s, m}$ denotes the (*s*, *m*)-th element of matrix *A*. $Col_{i}(A)$ denotes the *i*-th column of matrix *A*. $\mathscr {D}:=\{0, 1\}$, $\mathscr {D}^m:=\underbrace{\mathscr {D}\times \cdots \times \mathscr {D}}_{m}$. $\Delta _s:=\{\delta _s^i: i=1, \cdots , s\}$, where $\delta _s^i=Col_{i}(I_s)$. $A_{s\times m}$ is called a (*s*, *m*) logical matrix, if $Col_{i}(A_{s\times m})\in \Delta _s, i=1, \cdots , m$. ${\mathscr {L}}_{s\times m}$ denotes the set of $s\times m$ logical matrices. $W_{[s,m]}$ and $M_{r,n}$ are swap matrix and power-reducing matrix, respectively^8^. $[a, b)|_{\mathbb {Z}}=\{a, \cdots , b-1\}\subseteq \mathbb {Z}, \ a, b \in \mathbb {Z}, a<b$. $\lfloor c\rfloor$ represents the maximum integer not greater than *c*. Denote $a_{1}\widetilde{\bigwedge } a_{2}=\min \{a_{1}, a_{2}\}$. In this paper, the default matrix product is semi-tensor product ($\ltimes$)^8^.

## Problem formulation

Consider the following DBCN:

2 $$\begin{aligned} \left\{ \begin{array}{lll} x_{1}(t+1)=\kappa _{1}(X(t-\varsigma +1), \cdots , X(t), U(t)),\\ ~~~~\vdots \\ x_{n}(t+1)=\kappa _{n}(X(t-\varsigma +1), \cdots , X(t), U(t)),\end{array}\right. \end{aligned}$$

where $\varsigma \in \mathbb {Z_{+}}$ is the time delay, $X(i):=(x_{1}(i), \cdots , x_{n}(i))\in \mathscr {D}^n$, $i=-\varsigma +1,\cdots , 0, 1, \cdots$ denote the state, here $U(t):=(v_{1}(t), \cdots , v_{m}(t))\in \mathscr {D}^{m}$ denotes the control input, and $\kappa _{i}: \mathscr {D}^{n\varsigma +m} \rightarrow \mathscr {D}$, $i=1,\cdots ,n$ are Boolean functions. Assume that $Y_{0}:=(X(-\varsigma +1),\cdots ,X(0))\in \mathscr {D}^{n\varsigma }$ denotes the initial state trajectory.

Furthermore, consider state inequality constraints for binary variables $x_{j}(i)\in \mathscr {D}$, $j=1,\cdots ,n$:

3 $$\begin{aligned} a\le f(x_{1}(i), \cdots , x_{n}(i))\le b, \end{aligned}$$

where $f: \mathscr {D}^{n} \rightarrow \mathbb {R}$ is the inequality constrained function, and $a,b\in \mathbb {R}$ are inequality constrained boundaries.

For DBCN (2), we assume that $f(x_{1}(i), \cdots , x_{n}(i))$ satisfies the following linear form:

4 $$\begin{aligned} f(x_{1}(i), \cdots , x_{n}(i))=(2^{0}, 2^{1}, \cdots , 2^{n-1})(x_{1}(i), \cdots , x_{n}(i))^{\top }.\end{aligned}$$

It is worth pointing out that all possible states in $\mathscr {D}^n$ correspond one-to-one to indicator set $[0, 2^{n})|_{\mathbb {Z}}$, and some other types of inequalities (nonlinear) can be transformed into linear constraint forms of (4) through column expansion^35^.

**Fig. 1:**
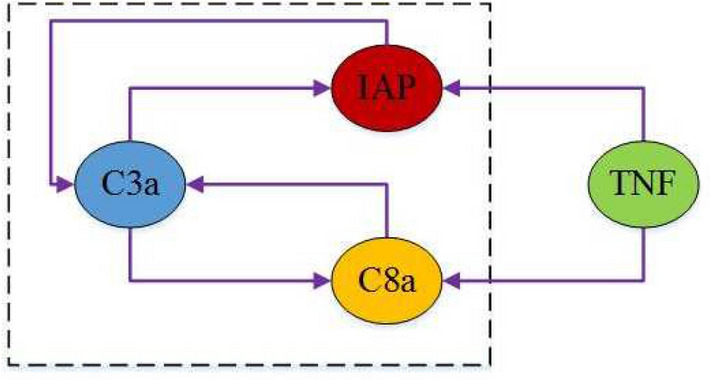
Network of the cell apoptosis network (5).

### *Example 1*

Consider the cell apoptosis network^36^

5 $$\begin{aligned} \left\{ \begin{array}{lll} IAP(t+1)=\lnot C3a(t)\wedge TNF(t),\\ C3a(t+1)=\lnot IAP(t)\wedge C8a(t),\\ C8a(t+1)=C3a(t)\vee TNF(t), \end{array}\right. \end{aligned}$$

where IAP, C3a, and C8a represent concentration levels (low or high) of apoptosis inhibitor protein, active cystatin 3, and active cystatin8, respectively; the concentration level of tumor necrosis factor (TNF) is considered as a control input. The network graph of (5) is shown in Fig. 1.

Set $$x_1(t)=IAP(t)$$, $$x_2(t)=C3a(t)$$, $$x_3(t)=C8a(t)$$, $$v(t)=TNF(t)$$, and

$$f(X(t))=(2^{0},2^{1},2^{2})(X(t))^{\top }=2^{0}x_{1}(t)+2^{1}x_{2}(t)+2^{2}x_{3}(t).$$

From $\mathscr {D}^3=\{(0, 0, 0),(1, 0, 0),(0, 1, 0),$ (1, 1, 0), (0, 0, 1),  (1, 0, 1),  (0, 1, 1),  $(1, 1, 1)\}$, the corresponding indicator can be obtained as $f(0, 0, 0)=0, f(1, 0, 0)=1,$ $f(0, 1, 0)=2, f(1, 1, 0)=3, f(0, 0, 1)=4, f(1, 0, 1)=5, f(0, 1, 1)=6,$ $f(1, 1, 1)=7.$

In practice, the concentration ratio of IAP, C3a, and C8a can only be effective within an indicator range, so only the data within the range needs to be considered. For example, the indicator range requires $2\le f(X(t))\le 5$, then the corresponding solution set is $C_{x}=\{(0, 1, 0),(1, 1, 0),(0, 0, 1)$, $(1, 0, 1)\}$, that is, the states of system (5) are constrained to $C_{x}.$

Firstly, we give the definitions of NSDC and nonuniform sampled-data inequality constrained set stabilization^5^.

### **Definition 1**

Given a set of sampling points $\{t_{h}: h\in \mathbb {N}\}$ with $t_0=0$. $\{U(t): t\in \mathbb {N}\}\subseteq \mathscr {D}^{m}$ is said to be an NSDC, if

6 $$\begin{aligned} U(t)=U(t_{h}), t\in [t_{h}, t_{h+1})| _{\mathbb {Z}}, t_{h+1}-t_{h}=\tau _{h}, \end{aligned}$$

where the interval length $\tau _{h}\in \mathbb {Z}_+$ between sampling points are time-variant.

Especially, when $\tau _{h}=\tau$ holds for any $h\in \mathbb {N}$, the definition of uniform SDC can be given, where $\tau \in \mathbb {Z}_+$ is called the sampling period^37^.

### **Definition 2**

Let a state inequality constraint (3) and a nonempty state set $\mathscr {E}_{e}$ that satisfies (3) be given. DBCN (2) is said to be nonuniform sampled-data inequality constrained set stabilizable to $\mathscr {E}_{e}$, if for any $Y_{0}$ that satisfies (3), there exist a time-variant state feedback NSDC

7 $$\begin{aligned} v_{i}(t)=g_{i}(t_{h}, X(t_{h}-\varsigma +1), \cdots ,X(t_{h})), t\in [t_{h}, t_{h+1})| _{\mathbb {Z}} \end{aligned}$$

with $g_{i}: \{t_{h}: h\in \mathbb {N}\}\times \mathscr {D}^{n\varsigma }\rightarrow \mathscr {D}, \ i=1,\cdots ,m$ being time-variant logical functions, and a positive integer *T* such that $X(t)\in \mathscr {E}_{e}, \forall \ t\ge T$, and $a\le f(X(t))\le b, \forall \ t \ge 1$.

Secondly, based on the ASSR^8^, we provide the equivalent algebraic form of DBCN (2).

Identifying $1\sim \delta _{2}^{1}$, $0\sim \delta _{2}^{2}$. Setting $x(t)=\ltimes _{i=1}^n x_i(t)\in \Delta _{2^n}$, $v(t)=\ltimes _{i=1}^m v_i(t)\in \Delta _{2^m}$, $y(t)=\ltimes _{i=t-\varsigma +1}^t x(i) \in \Delta _{2^{n\varsigma }}$, we can convert DBCN (2) into the algebraic form

8 $$\begin{aligned} \left\{ \begin{array}{lll} x_1(t+1)=K_{1}v(t)y(t),\\ ~~\vdots \\ x_n(t+1)=K_{n}v(t)y(t),\end{array}\right. \end{aligned}$$

where $K_{i}\in \mathscr {L}_{2\times {2^{n\varsigma +m}}}$ is the structural matrix of $\kappa _i$, $i=1,\cdots ,n$. Multiplying the *n* equations in (8), we can obtain the following form of (8):

9 $$\begin{aligned} x(t+1)=Kv(t)y(t), \end{aligned}$$

where $K\in \mathscr {L}_{2^n\times 2^{n\varsigma +m}}$ satisfies $Col_{j}(K)=\ltimes _{i=1}^{n}Col_{j}(K_{i})$, $j=1,\cdots ,2^{n\varsigma +m}$. For detailed instructions on how to use the ASSR to represent logical functions, please refer to^8^.

In addition, from (4) and the construction of $x(t)=\ltimes _{i=1}^n x_i(t)\in \Delta _{2^n}$, we can easily obtain the following result.

### Proposition 1

*If* $f(p_{1}, \cdots , p_{n})=p,\ (p_{1}, \cdots , p_{n})\in \mathscr {D}^n$, *then* $\delta _{2^{n}}^{p+1}=\delta _{2}^{p_{n}+1}\ltimes \cdots \ltimes \delta _{2}^{p_{1}+1}\in \Delta _{2^n}$ *holds*.

Based on Proposition 1, we present the following algorithm to determine the solution set for the state inequality constraint (3).

**Algorithm 1:**
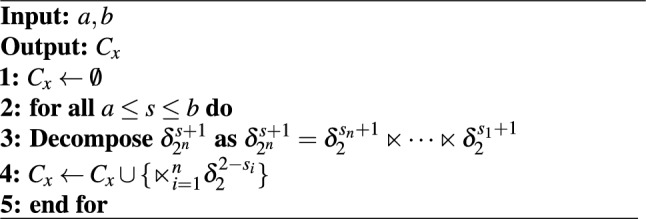
Construction of the solution set for the state inequality constraint (3).

Similar to DBCN (2), we can convert the NSDC (7) into the algebraic form:

10 $$\begin{aligned} v(t)=G(t_{h}) y(t_{h}), \ t\in [t_{h}, t_{h+1})| _{\mathbb {Z}}, \end{aligned}$$

where $G(t_{h})\in {\mathscr {L}}_{2^{m}\times 2^{n\varsigma }}$ is referred to as the time-variant state feedback sampled-data gain matrix.

In order to unify the dimensions of states in system (9), we convert system (9) into the augmented form

11 $$\begin{aligned} y(t+1)= & \ltimes _{i=t-\varsigma +2}^{t+1}x(i)\nonumber \\= & (\textbf{1}_{2^n}^{\top } \otimes I_{2^n})y(t)x(t+1) \nonumber \\= & (\textbf{1}_{2^n}^{\top } \otimes I_{2^n})y(t)Kv(t)y(t)\nonumber \\= & (\textbf{1}_{2^n}^{\top } \otimes I_{2^n})(I_{2^{n\varsigma }}\otimes K)W_{[2^{m},2^{n\varsigma }]}v(t)y^2(t)\nonumber \\= & (\textbf{1}_{2^n}^{\top } \otimes I_{2^n})(I_{2^{n\varsigma }}\otimes K)W_{[2^{m},2^{n\varsigma }]}(I_{2^m}\otimes M_{r,2^{n\varsigma }})v(t)y(t)\nonumber \\:= & \overline{K}v(t)y(t), \end{aligned}$$

where $$\overline{K}=(\textbf{1}_{2^n}^{\top } \otimes I_{2^n})(I_{2^{n\varsigma }}\otimes K)W_{[2^{m},2^{n\varsigma }]}(I_{2^m}\otimes M_{r,2^{n\varsigma }})\in \mathscr {L}_{{2^{n\varsigma }}\times {2^{n\varsigma +m}}}.$$

Assume $C_{x}=\{\delta _{2^n}^{\psi _{1}}, \cdots , \delta _{2^n}^{\psi _{\beta }}\}\subseteq \Delta _{2^{n}}$, where $\psi _{1}<\cdots <\psi _{\beta }$. Correspondingly, the states of (11) are constrained to

12 $$\begin{aligned} C_{y}=\{y=\ltimes _{i=1}^{\varsigma } x^{i} : x^{i}\in C_{x}\}:=\{\delta _{2^{n\varsigma }}^{\mu _{1}}, \cdots , \delta _{2^{n\varsigma }}^{\mu _{\beta ^{\varsigma }}}\},\end{aligned}$$

where $\mu _{1}<\cdots <\mu _{\beta ^{\varsigma }}$. For the nonempty set $\mathscr {E}_{e}\subseteq C_{x}$ in Definition 2, we denote

13 $$\begin{aligned} \widehat{\mathscr {E}}_{e}=\{y=\ltimes _{i=1}^{\varsigma } x^{i} : x^{i}\in \mathscr {E}_{e}\}\subseteq C_{y}. \end{aligned}$$

Finally, we study the relation between nonuniform sampled-data inequality constrained set stabilizable to $\mathscr {E}_{e}$ of DBCN (2) and nonuniform sampled-data inequality constrained set stabilizable to $\widehat{\mathscr {E}}_{e}$ of system (11). Before this, we first provide the definition of nonuniform sampled-data inequality constrained set stabilizable to $\widehat{\mathscr {E}}_{e}$ for (11).

### Definition 3

For the nonempty set $\widehat{\mathscr {E}}_{e}\subseteq C_{y}$, system (11) is said to be nonuniform sampled-data inequality constrained set stabilizable to $\widehat{\mathscr {E}}_{e}$, if $\forall \ y_{0} \in C_{y}$, there exist an NSDC (10) and an integer $\widehat{T}>0$ such that $y(t)\in \widehat{\mathscr {E}}_{e}, \forall \ t\ge \widehat{T}$, and $y(t)\in C_{y}, \forall \ t \ge 0.$

Then, we have the following result.

### Proposition 2

*DBCN* (2) *is nonuniform sampled-data inequality constrained set stabilizable to* $\mathscr {E}_{e}$, *if and only if system* (11) *is nonuniform sampled-data inequality constrained set stabilizable to* $\widehat{\mathscr {E}}_{e}.$

### *Proof*

(Necessity) From Definition 2, for any $y_{0}=\ltimes _{i=-\varsigma +1}^0 x(i)\in C_{y}$, there exist a time-variant state feedback NSDC sequence and a positive integer *T* such that $x(t)\in \mathscr {E}_{e},\ \forall \ t \ge T$, and $x(t)\in C_{x},\ \forall \ t\ge 1$, which implies $y(t)=\ltimes _{i=t-\varsigma +1}^t x(i) \in \widehat{\mathscr {E}}_{e}, \ \forall \ t \ge T+\varsigma -1$, and $y(t)=\ltimes _{i=t-\varsigma +1}^t x(i)\in C_{y}, \ \forall \ t\ge 1$. From Definition 3, system (11) is nonuniform sampled-data inequality constrained set stabilizable to $\widehat{\mathscr {E}}_{e}$.

(Sufficiency) From Definition 3, for any $y_{0}\in C_{y}$, there exist a time-variant state feedback NSDC sequence and a positive integer $\widehat{T}$ such that $y(t) \in \widehat{\mathscr {E}}_{e}, \ \forall \ t \ge \widehat{T}$, and $y(t)\in C_{y}$, $\forall \ t\ge 1$. From the construction of $\widehat{\mathscr {E}}_{e}$ and the unique factorization of $y(t)=\ltimes _{i=t-\varsigma +1}^t x(i)$, we have $x(t)\in \mathscr {E}_{e}$, $\forall \ t \ge \widehat{T}$, and $x(t)\in C_{x}$, $\forall \ t \ge 1$. From Definition 2, DBCN (2) is nonuniform sampled-data inequality constrained set stabilizable to $\mathscr {E}_{e}$. $\square$

## Inequality constrained set reachability

In this section, by constructing an inequality constrained controllability matrix, we investigate the nonuniform sampled-data inequality constrained set reachability of the augmented system (11).

Firstly, we give the concept of nonuniform sampled-data inequality constrained set reachability of system (11).

### Definition 4

Given a nonempty set $\widehat{\mathscr {E}}_{d}\subseteq C_{y}$ and an initial state $y_{0} \in C_{y}$. $\widehat{\mathscr {E}}_{d}$ is called nonuniform sampled-data inequality constrained set reachable from $y_{0}$ at sampling point $t_{h}$ under NSDC, if one can find an NSDC sequence $\big \{v(0),v(1),\cdots$ $,v(t_{h}-1)\big \}\subseteq \Delta _{2^{m}}$ such that $y(t_{h})\in \widehat{\mathscr {E}}_{d}$ and $y(t)\in C_{y}$, $\forall \ 1 \le t \le t_{h}$.

Secondly, we construct the inequality constrained controllability matrix.

For system (11), split $\overline{K}$ into $2^m$ equal blocks as

14 $$\begin{aligned} \overline{K}=[Blk_1(\overline{K})~\cdots ~Blk_{2^m}(\overline{K})]. \end{aligned}$$

Then, $Blk_i(\overline{K})$ corresponds to the control $\delta _{2^m}^i$, $i=1,\cdots ,2^m$. In order to reduce the computational complexity caused by state inequality constraints, define $E\in \mathbb {R}^{2^{n\varsigma }\times 2^{n\varsigma }}$ with

15 $$\begin{aligned} Row_{i}(E)=\left\{ \begin{array}{lll} (\delta _{2^{n\varsigma }}^{i})^{\top },\ \ i\in \{\mu _{1}, \cdots , \mu _{\beta ^{\varsigma }}\},\\ \textbf{0}_{2^{n\varsigma }}^{\top },\ \hbox {otherwise}.\end{array}\right. \end{aligned}$$

Let

16 $$\begin{aligned} Blk_i(\widehat{\overline{K}})=E \big (Blk_i(\overline{K})\big ) E^{\top }, i=1,\cdots ,2^m. \end{aligned}$$

Intuitively, $Blk_i(\widehat{\overline{K}})$ is obtained from $Blk_i(\overline{K})$ by substituting zeros in the corresponding rows and columns with indices $\{1,\cdots , {2^{n\varsigma }}\} \setminus \{\mu _{1}, \cdots , \mu _{\beta ^{\varsigma }}\}$. Then, the inequality constrained controllability matrix is constructed as follows:

17 $$\begin{aligned} Q_{\tau _{h}}=\sum _{i=1}^{2^{m}}\Big (Blk_{i}(\widehat{\overline{K}})\Big )^{\tau _{h}}, \end{aligned}$$

where $$\tau _{h}=t_{h+1}-t_{h}, h\in \mathbb {N}.$$

Finally, we present a criterion for the nonuniform sampled-data inequality constrained set reachability by (17).

### Theorem 1

*Given a nonempty set* $\widehat{\mathscr {E}}_{d}\subseteq C_{y}$ *and an initial state* $y(0)=\delta _{2^{n\varsigma }}^{\mu _{\theta }}\in C_{y}$. $\widehat{\mathscr {E}}_{d}$ *is nonuniform sampled-data inequality constrained set reachable from* *y(0) at sampling* *point* $t_{h}$ *under NSDC, if and only if*

18 $$\begin{aligned} \sum _{\delta _{2^{n\varsigma }}^{\mu _{h_{i}}}\in \widehat{\mathscr {E}}_{d}}(Q_{\tau _{h-1}}\cdots Q_{\tau _{0}})_{\mu _{h_{i}},\mu _{\theta }}\ge 1. \end{aligned}$$

### *Proof*

(Necessity) Assuming that $\widehat{\mathscr {E}}_{d}$ is inequality constrained set reachable from $y(0)=\delta _{2^{n\varsigma }}^{\mu _{\theta }}$ at $t_{h}$ under NSDC, we prove (18) by induction.

For $h=1$, from Definition 4, there exist $v(0)=\delta _{2^{m}}^{\xi _{0}}$, $\cdots ,v(t_{1}-1)=\delta _{2^{m}}^{\xi _{0}}$ and $y(t_{1})=\delta _{2^{n\varsigma }}^{\mu _{{\tilde{1}}_{i}}}\in \widehat{\mathscr {E}}_{d}$ such that $\delta _{2^{n\varsigma }}^{\mu _{{\tilde{1}}_{i}}}=\Big (Blk_{\xi _{0}}(\widehat{\overline{K}})\Big )^{\tau _{0}}$ $\delta _{2^{n\varsigma }}^{\mu _{\theta }}$ and $y(t)\in C_{y}$, $\forall \ 1 \le t \le t_{1}$. Thus, $\Big (\big (Blk_{\xi _{0}}(\widehat{\overline{K}})\big )^{\tau _{0}}\Big )_{\mu _{{\tilde{1}}_{i}},\mu _{\theta }}=1$, which shows that

19 $$\begin{aligned} \sum _{\delta _{2^{n\varsigma }}^{\mu _{1_{i}}}\in \widehat{\mathscr {E}}_{d}}(Q_{\tau _{0}})_{\mu _{1_{i}},\mu _{\theta }} \ge \Big (\sum _{j=1}^{2^{m}}\big (Blk_{j}(\widehat{\overline{K}})\big )^{\tau _{0}}\Big )_{\mu _{{\tilde{1}}_{i}},\mu _{\theta }} \ge \Big (\big (Blk_{\xi _{0}}(\widehat{\overline{K}})\big )^{\tau _{0}}\Big )_{\mu _{{\tilde{1}}_{i}},\mu _{\theta }}=1, \end{aligned}$$

that is, (18) holds for $h=1.$

Assume that (18) holds for some $h=\lambda >1$, that is

20 $$\begin{aligned} \sum _{\delta _{2^{n\varsigma }}^{\mu _{\lambda _{i}}}\in \widehat{\mathscr {E}}_{d}}(Q_{\tau _{\lambda -1}}\cdots Q_{\tau _{0}})_{\mu _{\lambda _{i}},\mu _{\theta }}\ge 1.\end{aligned}$$

Then, there exist $v(t)|_{t=t_{0}}^{t_{1}-1}=\delta _{2^{m}}^{\xi _{0}}, \cdots , v(t)|_{t=t_{\lambda -1}}^{t_{\lambda }-1}=\delta _{2^{m}}^{\xi _{\lambda -1}}$ and $y(t_{\lambda })=\delta _{2^{n\varsigma }}^{\mu _{{\tilde{\lambda}}_{i}}}\in \widehat{\mathscr {E}}_{d}$ such that

21 $$\begin{aligned} \Big (\big (Blk_{\xi _{\lambda -1}}(\widehat{\overline{K}})\big )^{\tau _{\lambda -1}} \cdots \big (Blk_{\xi _{0}}(\widehat{\overline{K}})\big )^{\tau _{0}}\Big )_{\mu _{{\tilde{\lambda}}_{i}},\mu _{\theta }}=1, \end{aligned}$$

where $v(t)|_{t=t_{h}}^{t_{h+1}-1}=\delta _{2^m}^{\xi _{h}}$ denotes an NSDC sequence $\{v(t_{h})=\delta _{2^m}^{\xi _{h}},\cdots ,v(t_{h+1}-1)=\delta _{2^m}^{\xi _{h}}\}\subseteq \Delta _{2^{m}}$, $h\in \mathbb {N}$.

We prove that (18) holds for $h=\lambda +1$. By (21) and Definition 4, there exist $v(t)|_{t=t_{0}}^{t_{1}-1}=\delta _{2^{m}}^{\xi _{0}}, \ \cdots ,\ v(t)|_{t=t_{\lambda -1}}^{t_{\lambda }-1}=\delta _{2^{m}}^{\xi _{\lambda -1}},$ $v(t)|_{t=t_{\lambda }}^{t_{\lambda +1}-1}$ $=\delta _{2^{m}}^{\xi _{\lambda }}$ and $y(t_{\lambda +1})=\delta _{2^{n\varsigma }}^{\mu _{\widetilde{(\lambda +1)}_{i}}}\in \widehat{\mathscr {E}}_{d}$ such that the trajectory from $\delta _{2^{n\varsigma }}^{\mu _{\theta }}$ to $\delta _{2^{n\varsigma }}^{\mu _{\widetilde{(\lambda +1)}_{i}}}$ can be decomposed to the trajectory from $\delta _{2^{n\varsigma }}^{\mu _{\theta }}$ to

22 $$\begin{aligned} y(t_{\lambda }) =\Big (\big (Blk_{\xi _{\lambda -1}}(\widehat{\overline{K}})\big )^{\tau _{\lambda -1}} \cdots \big (Blk_{\xi _{0}}(\widehat{\overline{K}})\big )^{\tau _{0}}\Big )\delta _{2^{n\varsigma }}^{\mu _{\theta }} =\delta _{2^{n\varsigma }}^{\mu _{{\tilde{\lambda}}_{i}}}\in C_{y} \end{aligned}$$

at sampling point $t_{\lambda }$ and the trajectory from $\delta _{2^{n\varsigma }}^{\mu _{{\tilde{\lambda}}_{i}}}$ to $\delta _{2^{n\varsigma }}^{\mu _{\widetilde{(\lambda +1)}_{i}}}$ in $\tau _{\lambda }$ steps. Then, from (19) and (22), we have

$$\begin{aligned} \Big (\big (Blk_{\xi _{\lambda }}(\widehat{\overline{K}})\big )^{\tau _{\lambda }}\Big ) _{\mu _{\widetilde{(\lambda +1)}_{i}},\mu _{{\tilde{\lambda}}_{i}}}=1 \quad \text {and}\quad \Big (\big (Blk_{\xi _{\lambda -1}}(\widehat{\overline{K}})\big )^{\tau _{\lambda -1}} \cdots \big (Blk_{\xi _{0}}(\widehat{\overline{K}})\big )^{\tau _{0}}\Big )_{\mu _{{\tilde{\lambda}}_{i}},\mu _{\theta }}=1, \end{aligned}$$

which show that

$$\begin{aligned}&\sum _{\delta _{2^{n\varsigma }}^{\mu _{(\lambda +1)_{i}}}\in \widehat{\mathscr {E}}_{d}}(Q_{\tau _{\lambda }}Q_{\tau _{\lambda -1}}\cdots Q_{\tau _{0}})_{\mu _{(\lambda +1)_{i}},\mu _{\theta }} \ge \sum ^{2^{n\varsigma }}_{j=1}(Q_{\tau _{\lambda }})_{\mu _{\widetilde{(\lambda +1)}_{i}},j} (Q_{\tau _{\lambda -1}}\cdots Q_{\tau _{0}})_{j,\mu _{\theta }}\\&\quad \ge \Big (\big (Blk_{\xi _{\lambda }}(\widehat{\overline{K}})\big )^{\tau _{\lambda }} \Big )_{\mu _{\widetilde{(\lambda +1)}_{i}},\mu _{{\tilde{\lambda}}_{i}}} \Big (\big (Blk_{\xi _{\lambda -1}}(\widehat{\overline{K}})\big )^{\tau _{\lambda -1}} \cdots \big (Blk_{\xi _{0}}(\widehat{\overline{K}})\big )^{\tau _{0}}\Big )_{\mu _{{\tilde{\lambda}}_{i}},\mu _{\theta }} =1. \end{aligned}$$

Thus, (18) holds for $h=\lambda +1.$ By induction, the necessity is proved.

(Sufficiency) Assume that (18) holds, that is,

$$\begin{aligned} \sum _{\delta _{2^{n\varsigma }}^{\mu _{h_{i}}}\in \widehat{\mathscr {E}}_{d}}(Q_{\tau _{h-1}}\cdots Q_{\tau _{0}})_{\mu _{h_{i}},\mu _{\theta }}=\sum _{\delta _{2^{n\varsigma }}^{\mu _{h_{i}}}\in \widehat{\mathscr {E}}_{d}}\Big (\sum _{j=1}^{2^{m}}\big (Blk_{j}(\widehat{\overline{K}})\big )^{\tau _{h-1}}\cdots \sum _{j=1}^{2^{m}}\big (Blk_{j}(\widehat{\overline{K}})\big )^{\tau _{0}}\Big )_{\mu _{h_{i}},\mu _{\theta }} \ge 1. \end{aligned}$$

Then, there exist $v(t)|_{t=t_{0}}^{t_{1}-1}=\delta _{2^{m}}^{\xi _{0}}, \cdots , v(t)|_{t=t_{h-1}}^{t_{h}-1}=\delta _{2^{m}}^{\xi _{h-1}}$ and $y(t_{h})=\delta _{2^{n\varsigma }}^{\mu _{\widetilde{h}_{i}}}\in \widehat{\mathscr {E}}_{d}$ such that

$$\begin{aligned} \Big (\big (Blk_{\xi _{h-1}}(\widehat{\overline{K}})\big )^{\tau _{h-1}}\cdots \big (Blk_{\xi _{0}}(\widehat{\overline{K}})\big )^{\tau _{0}}\Big )_{\mu _{\widetilde{h}_{i}},\mu _{\theta }}=1. \end{aligned}$$

Thus, $$y(t_{h})=\delta _{2^{n\varsigma }}^{\mu _{\widetilde{h}_{i}}}=\Big (\big (Blk_{\xi _{h-1}}(\widehat{\overline{K}}) \big )^{\tau _{h-1}}\cdots \big (Blk_{\xi _{0}}(\widehat{\overline{K}})\big )^{\tau _{0}}\Big )\delta _{2^{n\varsigma }}^{\mu _{\theta }} \in \widehat{\mathscr {E}}_{d}.$$

Finally, we prove $y(t)\in C_{y}, \ \forall \ 1 \le t \le t_{h}$ by reduction to absurdity. In fact, if there exists $1 \le t^{\prime }< t_{h}$ satisfying $y(t^{\prime })\notin C_{y}$, then by (16), $y(t)=\textbf{0}_{2^{n\varsigma }}$ for any $t^{\prime }< t\le t_{h}$, which is a contradiction to $y(t_{h}) \in \widehat{\mathscr {E}}_{d}.$

Thus, from Definition 4, $\widehat{\mathscr {E}}_{d}$ is inequality constrained set reachable from *y*(0) at $t_{h}$ under NSDC. $\square$

### *Example 2*

Consider DBCN (2) with equivalent algebraic form (11), where $\varsigma =1$, $C_{x}=\Delta _{2^{3}}$, and $\overline{K}=\delta _{8}[1 ~ 7 ~ 8~ 3 ~ 4 ~ 5~ 7 ~ 6~3 ~ 6~ 3 ~ 2$ 4 6 7 8]. Assume $\widehat{\mathscr {E}}_{d}=\{\delta _8^7\}$, $y(0)=\delta _{8}^1.$

(i) Suppose $\tau _{h}=1, \forall \ h\in \mathbb {N}$. According to Theorem 1, $\widehat{\mathscr {E}}_{d}$ is inequality constrained set reachable from $\delta _{8}^1$ at time $t=7$ under the traditional state feedback control sequence $\{v(0)=\delta _{2}^2,v(1)=\delta _{2}^1,v(2)=\delta _{2}^1,v(3)=\delta _{2}^1,v(4)=\delta _{2}^2,v(5)=\delta _{2}^2, v(6)=\delta _{2}^1\}$;
(ii) Suppose $\tau _{h}=\tau =2, \forall \ h\in \mathbb {N}$, where $\tau =2$ is the sampling period. According to Theorem 1, $\widehat{\mathscr {E}}_{d}$ is unreachable from $\delta _{8}^1$ under any state feedback uniform SDC sequence;
(iii) Suppose $\tau _{0}=2,\tau _{1}=4,\tau _{2}=1,\tau _{3}=2,\cdots$. According to Theorem 1, $\widehat{\mathscr {E}}_{d}$ is inequality constrained set reachable from $\delta _{8}^1$ at sampling point $t_{4}$ under the state feedback NSDC sequence $\{v(t)|_{t=t_{0}}^{t_{1}-1}=\delta _{2}^2,v(t)|_{t=t_{1}}^{t_{2}-1}=\delta _{2}^1,v(t)|_{t=t_{2}}^{t_{3}-1}= \delta _{2}^2,v(t)|_{t=t_{3}}^{t_{4}-1}=\delta _{2}^1\}$.

**Fig. 2:**
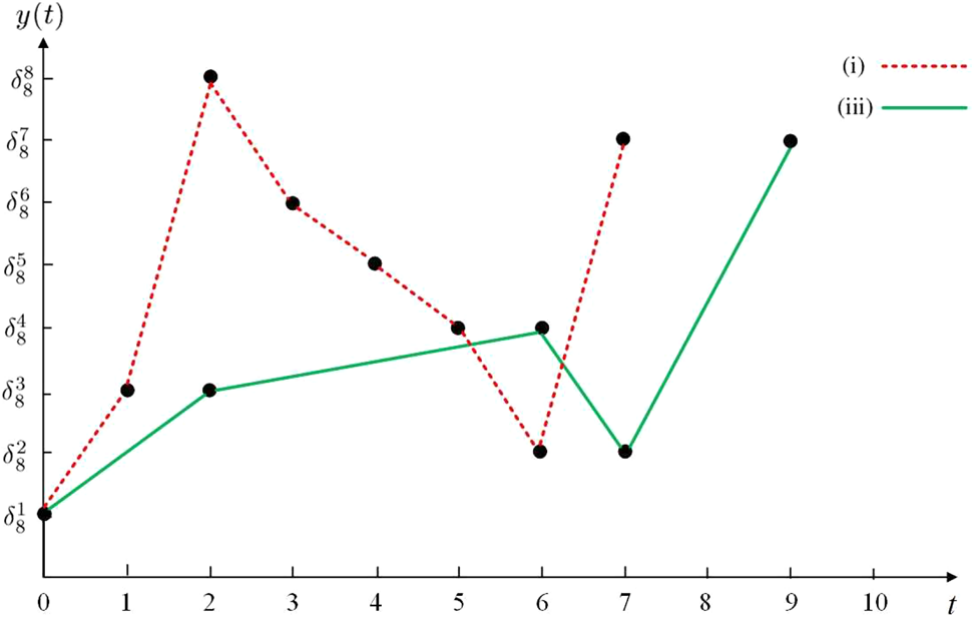
State trajectories in (i) and (iii).

### *Remark 1*

Traditional state feedback control^15^ and state feedback uniform SDC^37^ can be viewed as a special case of NSDC. NSDC can provide us with more control schemes to achieve control objectives and reduce control costs (see Fig. 2).

## Inequality constrained set stabilization

Based on the inequality constrained set reachability, we investigate the nonuniform sampled-data inequality constrained set stabilization.

Firstly, we introduce the concept of the largest nonuniform sampled-data inequality constrained invariant subset.

### Definition 5

A nonempty subset $\mathscr {E}^{\prime }\subseteq C_{y}$ is referred to as a nonuniform sampled-data inequality constrained invariant subset, if for any $y(0)\in \mathscr {E}^{\prime }$, one can find a state feedback NSDC $v(t_{0})\in \Delta _{2^{m}}$ such that $y(t)\in \mathscr {E}^{\prime }$ holds for any $t \le \overline{\tau }$, $\overline{\tau }=\max \{\tau _{h}, h\in \mathbb {N}\}.$

From Definition 5, the union of inequality constrained invariant subsets is still an inequality constrained invariant subset.

### Definition 6

Given a nonempty set $\mathscr {E}\subseteq C_{y}$, $I(\mathscr {E})$ is said to be the largest nonuniform sampled-data inequality constrained invariant subset of $\mathscr {E}$, if $I(\mathscr {E})$ is the union of all nonuniform sampled-data inequality constrained invariant subsets contained in $\mathscr {E}.$

From Definition 6, the construction method of the largest nonuniform sampled-data inequality constrained invariant subset $I(\mathscr {E})$ is given as fellow:

(i) Set $$\Gamma _{0}:=\{\mu :\delta _{2^{n\varsigma }}^\mu \in \mathscr {E}\}$$;
(ii) Set $$\Gamma _{j}:=\Big \{\mu \in \Gamma _{j-1}:\ \hbox {there exists an integer}$$ $$\ 0< \xi _{\mu }\le 2^{m},\ \hbox {such that}\widetilde{\bigwedge }_{l=1}^{\overline{\tau }}\Big (\sum _{i\in \Gamma _{j-1}}\big (\big (Blk_{\xi _{\mu }}(\widehat{\overline{K}})\big )^{l}\big )_{i,\mu }\Big )=1\Big \}, \ j\in \mathbb {Z}_{+};$$
(iii) Find the smallest positive integer $q\le |\mathscr {E}|$ such that $\Gamma _{q}=\Gamma _{q+1}$;
(iv) $$I(\mathscr {E})=\{\delta _{2^{n\varsigma }}^\mu :\mu \in \Gamma _{q}\}$$.

Secondly, given $\chi \in \mathbb {Z}_{+}$, we define the nonuniform sampled-data inequality constrained reachable sets as

$$\begin{aligned}&E_{\tau _{\chi -1}}(I(\widehat{\mathscr {E}}_{e}))=\Big \{\delta _{2^{n\varsigma }}^{\alpha }\in C_{y}:\hbox {there exists an integer} \ 1\le \xi _{\alpha }\\&\quad \le 2^{m}, \hbox {such that}\sum _{\delta _{2^{n\varsigma }}^{\mu _{\chi _{i}}}\in I(\widehat{\mathscr {E}}_{e})}\big (\big (Blk_{\xi _{\alpha }}(\widehat{\overline{K}})\big )^{\tau _{\chi -1}}\big )_{\mu _{\chi _{i}},\alpha }=1\Big \}, \end{aligned}$$

and

$$\begin{aligned}&E_{\tau _{\chi -2}+\tau _{\chi -1}}(I(\widehat{\mathscr {E}}_{e}))\\&\quad =\Big \{\delta _{2^{n\varsigma }}^{\alpha }\in C_{y}:\hbox {there exists an integer} 1\le \xi _{\alpha }\\&\quad \le 2^{m},\ \hbox {such that}\sum _{\delta _{2^{n\varsigma }}^{\mu _{(\chi -1)_{i}}}\in E_{\tau _{\chi -1}}(I(\widehat{\mathscr {E}}_{e}))}\big (\big (Blk_{\xi _{\alpha }}(\widehat{\overline{K}})\big )^{\tau _{\chi -2}}\big ) _{\mu _{(\chi -1)_{i}},\alpha }=1\Big \}. \end{aligned}$$

Keeping this procedure going, we define

$$\begin{aligned} & E_{\Sigma _{i=0}^{\chi -1}\tau _{i}}(I(\widehat{\mathscr {E}}_{e}))=\Big \{\delta _{2^{n\varsigma }}^{\alpha }\in C_{y}:\hbox {there exists an integer} 1\le \xi _{\alpha }\\ & \quad \le 2^{m},\ \hbox {such that}\sum _{\delta _{2^{n\varsigma }}^{\mu _{1_{i}}}\in E_{\Sigma _{i=1}^{\chi -1}\tau _{i}}(I(\widehat{\mathscr {E}}_{e}))}\big (\big (Blk_{\xi _{\alpha }}(\widehat{\overline{K}})\big )^{\tau _{0}}\big ) _{\mu _{1_{i}},\alpha }=1\Big \}. \end{aligned}$$

Thus, by the construction process of the largest nonuniform sampled-data inequality constrained invariant subset, if $I(\widehat{\mathscr {E}}_{e})\ne \emptyset$, $\delta _{2^{n\varsigma }}^{\alpha }\in E_{\Sigma _{i=0}^{\chi -1}\tau _{i}}(I(\widehat{\mathscr {E}}_{e}))$, then $\delta _{2^{n\varsigma }}^{\alpha }\in E_{\Sigma _{i=0}^{q}\tau _{i}}(I(\widehat{\mathscr {E}}_{e})), \forall \ q\ge \chi -1$. Hence, we have the following result on the inequality constrained reachable sets.

### Proposition 3

*If* $I(\widehat{\mathscr {E}}_{e})\ne \emptyset$, *then* $E_{\Sigma _{i=0}^{\chi -1}\tau _{i}}(I(\widehat{\mathscr {E}}_{e})) \subseteq E_{\Sigma _{i=0}^{\chi }\tau _{i}}(I(\widehat{\mathscr {E}}_{e}))$ *holds for any* $\chi \in \mathbb {Z}_{+}.$

Finally, based on the inequality constrained reachable sets, we provide the result on the inequality constrained set stabilization.

### Theorem 2

*System* (11) *is nonuniform sampled-data inequality constrained set stabilizable to* $\widehat{\mathscr {E}}_{e}$ *under a time-variant state feedback NSDC* (10), *if and only if*

(i) $$I(\widehat{\mathscr {E}}_{e})\ne \emptyset$$;
(ii) there exists an integer $1\le \chi \le \beta ^{\varsigma }$ such that $E_{\Sigma _{i=0}^{\chi -1}\tau _{i}}(I(\widehat{\mathscr {E}}_{e}))=C_{y}$.

### *Proof*

(Necessity) Obviously, from Definition 3, (i) holds. Now, we prove (ii).

From Definition 3, there exist an integer $T > 0$ and a time-variant state feedback NSDC such that

23 $$\begin{aligned} y(t)\in I(\widehat{\mathscr {E}}_{e}), \forall \ t\ge T, \ \forall \ y(0)\in C_{y}. \end{aligned}$$

Take $\hat{T}$ to represent the smallest integer $T>0$ that satisfies (23). By reduction to absurdity, we prove $\hat{T}<\beta ^{\varsigma }\overline{\tau }$. If $\hat{T}\ge \beta ^{\varsigma }\overline{\tau }$, we have $y(t_{h})\notin I(\widehat{\mathscr {E}}_{e}), h=0,\cdots ,\beta ^{\varsigma }$. However, since system (11) with the state inequality constraint has at most $\beta ^{\varsigma }$ different states, there exist different $h_{1}, h_{2}\in \{0,1,\cdots ,\beta ^{\varsigma }\}$ such that $y(t_{h_{1}})=y(t_{h_{2}})$. Hence, under the NSDC, starting from $y^{\prime }(0)=y(t_{h_{1}})$, the state trajectory forms a cycle, which contradicts the fact that system (11) is inequality constrained set stabilizable to $\widehat{\mathscr {E}}_{e}$ under the NSDC.

Setting $\chi =\lfloor \hat{T}/\overline{\tau }\rfloor +1\le \beta ^{\varsigma }$, we have $E_{\sum ^{\chi -1}_{i=0}\tau _{i}}(I(\widehat{\mathscr {E}}_{e}))=C_{y}$. Thus, (ii) holds.

(Sufficiency) Assume that (i) and (ii) hold. For each $y(0)=\delta _{2^{n\varsigma }}^{\alpha }\in E_{\Sigma _{i=0}^{\chi -1}\tau _{i}}(I$ $(\widehat{\mathscr {E}}_{e}))=C_{y}$, there exists an integer $0<\xi _{\alpha }\le 2^{m}$ such that $E_{\Sigma _{i=1}^{\chi -1}\tau _{i}}(I(\widehat{\mathscr {E}}_{e}))$ is inequality constrained set reachable from $\delta _{2^{n\varsigma }}^{\alpha }$ in $\tau _{0}$ steps under the NSDC $v(t_{0})=\delta _{2^{m}}^{\xi _{\alpha }}$. Set $G(t_{0})=\delta _{2^{m}}[\eta _{1}^{t_{0}}~\cdots ~\eta _{2^{n\varsigma }}^{t_{0}}]$, where

24 $$\begin{aligned} \eta _{i}^{t_{0}}\in \left\{ \begin{array}{lll} \{\xi _{\alpha }\}, \ \hbox {if} \ i=\alpha , \alpha =\mu _{1}, \cdots ,\mu _{\beta ^{\varsigma }} ,\\ \{1,\cdots , 2^{m}\},\ \hbox {otherwise}. \end{array} \right. \end{aligned}$$

Under the NSDC $v(t_{0})=G(t_{0})y(0)$, let

$$\begin{aligned} \Upsilon _{\tau _{0}}(y(0))&=\Big \{y(t_{1})\big |y(t_{1})=(\overline{K}v(t_{0}))^{\tau _{0}}y(0)\in C_{y}, y(0)\in E_{\sum ^{\chi -1}_{i=0}\tau _{i}}(\widehat{\mathscr {E}}_{e})\Big \}\\&:=\Big \{\delta _{2^{n\varsigma }}^{\alpha _{1}^{1}},\cdots ,\delta _{2^{n\varsigma }}^{\alpha _{\varrho _{1}}^{1}}\Big \} \subseteq E_{\Sigma _{i=1}^{\chi -1}\tau _{i}}(I(\widehat{\mathscr {E}}_{e})). \end{aligned}$$

For each $\delta _{2^{n\varsigma }}^{\alpha _{j}^{1}}\in \Upsilon _{\tau _{0}}(y(0))$, $j=1,\cdots ,\varrho _{1}$, there exists an integer $1\le \xi _{\alpha _{j}^{1}}\le 2^{m}$ such that $E_{\Sigma _{i=2}^{\chi -1}\tau _{i}}(I(\widehat{\mathscr {E}}_{e}))$ is inequality constrained set reachable from $y(t_{1})=\delta _{2^{n\varsigma }}^{\alpha _{j}^{1}}$ in $\tau _{1}$ steps under the NSDC $v(t_{1})=\delta _{2^{m}}^{\xi _{\alpha _{j}^{1}}}$. Set $G(t_{1})=\delta _{2^{m}}[\eta _{1}^{t_{1}}~\cdots ~\eta _{2^{n\varsigma }}^{t_{1}}]$, where

25 $$\begin{aligned} \eta _{i}^{t_{1}}\in \left\{ \begin{array}{lll} \{\xi _{\alpha _{j}^{1}}\}, \ \hbox {if} \ i=\alpha _{j}^{1}, \ j=1,\cdots ,\varrho _{1},\\ \{1,\cdots , 2^{m}\},\ \hbox {otherwise}. \end{array} \right. \end{aligned}$$

Under the NSDC $v(t_{1})=G(t_{1})y(t_{1})$, let

$$\begin{aligned} \Upsilon _{\tau _{1}+\tau _{0}}(y(0))&=\Big \{y(t_{2})\big |y(t_{2})=(\overline{K}v(t_{1}))^{\tau _{1}}y(t_{1})\in C_{y}, y(t_{1})\in \Upsilon _{\tau _{0}}(y(0))\Big \}\\&:=\Big \{\delta _{2^{n\varsigma }}^{\alpha _{1}^{2}},\cdots ,\delta _{2^{n\varsigma }}^{\alpha _{\varrho _{2}}^{2}}\Big \} \subseteq E_{\Sigma _{i=2}^{\chi -1}\tau _{i}}(I(\widehat{\mathscr {E}}_{e})). \end{aligned}$$

Keeping this procedure going, we have $G(t_{i})=\delta _{2^{m}}[\eta _{1}^{t_{i}}~\cdots ~\eta _{2^{n\varsigma }}^{t_{i}}], \ i=0,\cdots ,$ $\chi -1$. Under the NSDC

$$\begin{aligned} v(t_{\chi -1})=G(t_{\chi -1})y(t_{\chi -1}), \end{aligned}$$

let

$$\begin{aligned} \Upsilon _{\Sigma _{i=0}^{\chi -1}\tau _{i}}(y(0)) =\Big \{y(t_{\chi })\big |y(t_{\chi })=(\overline{K}v(t_{\chi -1}))^{\tau _{\chi -1}}y(t_{\chi -1})\in C_{y}, y(t_{\chi -1})\in \Upsilon _{\Sigma _{i=0}^{\chi -2}\tau _{i}}(y(0))\Big \} \subseteq I(\widehat{\mathscr {E}}_{e}). \end{aligned}$$

From (i) and the largest nonuniform sampled-data inequality constrained invariant subset, for each $\delta _{2^{n\varsigma }}^{\alpha }\in I(\widehat{\mathscr {E}}_{e})$, there exists an integer $0<\xi _{\alpha }\le 2^{m}$ such that $y(t)=(\overline{K}\delta _{2^{m}}^{\xi _{\alpha }})^{t-t_{h}}\delta _{2^{n\varsigma }}^{\alpha }\in I(\widehat{\mathscr {E}}_{e}), \forall \ t_{h}\le t\le t_{h+1}, \forall \ h\ge \chi .$ Set $G(t_{h})=\delta _{2^{m}}[\eta _{1}^{t_{\chi }}~\cdots ~\eta _{2^{n\varsigma }}^{t_{\chi }}]$, where

26 $$\begin{aligned} \eta _{i}^{t_{\chi }}\in \left\{ \begin{array}{lll} \{\xi _{\alpha }\}, \ \hbox {if} \ i=\alpha ,\\ \{1,\cdots , 2^{m}\},\ \hbox {otherwise}. \end{array} \right. \end{aligned}$$

For each $y(t_{h})\in I(\widehat{\mathscr {E}}_{e})$, under the NSDC $v(t_{h})=\delta _{2^{m}}[\eta _{1}^{t_{\chi }}~\cdots ~\eta _{2^{n\varsigma }}^{t_{\chi }}]y(t_{h})$, we have $y(t)\in I(\widehat{\mathscr {E}}_{e}), \forall \ t_{h}\le t\le t_{h+1},\ h\ge \chi .$

Thus, we obtain the time-variant state feedback NSDC as follows:

27 $$\begin{aligned} v(t) =G(t_{h})y(t_{h}) =\left\{ \begin{array}{lll} \delta _{2^{m}}[\eta _{1}^{t_{h}}~\cdots ~\eta _{2^{n\varsigma }}^{t_{h}}]y(t_{h}), \ h=0,\cdots ,\chi -1,\\ \delta _{2^{m}}[\eta _{1}^{t_{\chi }}~\cdots ~\eta _{2^{n\varsigma }}^{t_{\chi }}]y(t_{h}), \ h\ge \chi . \end{array} \right. \end{aligned}$$

$t\in [t_{h}, t_{h+1})| _{\mathbb {Z}}$, under which system (11) is nonuniform sampled-data inequality constrained set stabilizable to $\widehat{\mathscr {E}}_{e}$. $\square$

### Corollary 1

*For* $\mathscr {E}_{e}\subseteq C_{x}$ *given in Definition* 2, *DBCN* (2) *is nonuniform sampled-data inequality constrained set stabilizable to* $\mathscr {E}_{e}$ *by a time-variant state feedback NSDC* (7), *if and only if (i) and (ii) of Theorem* 2*hold*.

### *Remark 2*

The state constraints in BNs are generally directly given a state constraint set, while state inequality constraints require solving the state constraint set based on the constraints satisfied by the state. Meanwhile, this paper provides a method for determining the state constraint set based on the state inequality constraints. This provides technical support for studying the stabilization problem of BNs under different constraint conditions.

## Illustrative example

### *Example 3*

Consider the apoptosis network (5):

28 $$\begin{aligned} \left\{ \begin{array}{lll} x_{1}(t+1)=\lnot x_2(t-1)\wedge v(t),\\ x_{2}(t+1)=\lnot x_1(t-1)\wedge x_3(t-1),\\ x_{3}(t+1)= x_2(t-1)\vee v(t), \end{array}\right. \end{aligned}$$

with the state time delay $\varsigma =2$ and the state inequality constraint

29 $$\begin{aligned} 2\le 2^{0}x_{1}(i)+2^{1}x_{2}(i)+2^{2}x_{3}(i)\le 4, \ i=-1, 0, 1, \cdots . \end{aligned}$$

Setting $x(t)=\ltimes _{i=1}^3 x_i(t)$, $y(t)=x(t-1)\ltimes x(t)$, from system (11), we have

30 $$\begin{aligned} y(t+1)=\overline{K}v(t)y(t), \end{aligned}$$

where

$$\begin{aligned} \overline{K}=\delta _{64}[&7~15~23~31~39~47~55~63~7~15~23~31~39~47~55~63~3~11~19~27~35~43~51~59~3~11~19~27~35~43~51~59\\&5~13~21~29~37~45~53~61~7~15~23~31~39~47~55~63~1~~9~17~25~33~41~49~57~3~11~19~27~35~43~51~59\\&7~15~23~31~39~47~55~63~7~15~23~31~39~47~55~63~8~16~24~32~40~48~56~64~8~16~24~32~40~48~56~64\\&5~13~21~29~37~45~53~61~7~15~23~31~39~47~55~63~6~14~22~30~38~46~54~62~8~16~24~32~40~48~56~64]. \end{aligned}$$

By Algorithm 1, we have $C_{x}=\{\delta _{8}^{2}, \delta _{8}^{6}, \delta _{8}^{7}\}.$

Next, we study the nonuniform sampled-data inequality constrained set stabilization of system (28) with $\mathscr {E}_{e}=\{\delta _{8}^{6}, \delta _{8}^{7}\}$

and

31 $$\begin{aligned} \tau _{h}=\left\{ \begin{array}{lll} 2, \ h=2i,\\ 1,\ h=2i+1, \end{array} \right. i\in \mathbb {N}. \end{aligned}$$

From (12) and (13), we have $C_{y}=\{\delta _{64}^{10}, \delta _{64}^{14}, \delta _{64}^{15}, \delta _{64}^{42}$, $\delta _{64}^{46}, \delta _{64}^{47}, \delta _{64}^{50}, \delta _{64}^{54}, \delta _{64}^{55}\}$ and $\widehat{\mathscr {E}}_{e}=\{\delta _{64}^{46}, \delta _{64}^{47}, \delta _{64}^{54}, \delta _{64}^{55}\}.$

By the proof process of Theorem 1, we have $I(\widehat{\mathscr {E}}_{e})=\widehat{\mathscr {E}}_{e}$ and $E_{\tau _{0}}(I(\widehat{\mathscr {E}}_{e}))=C_{y}$. From Corollary 1, system (28) is inequality constrained set stabilizable to $\mathscr {E}_{e}$. In addition, by Theorem 2, the time-variant state feedback sampled-data gain matrix is designed as

32 $$\begin{aligned} G(t_{0})=\delta _{2}[\eta _{1}^{t_{0}}~\cdots ~\eta _{64}^{t_{0}}], \ G(t_{h})=\delta _{2}[\eta _{1}^{t_{h}}~\cdots ~\eta _{64}^{t_{h}}], \end{aligned}$$

where

$$\begin{aligned} \eta _{i}^{t_{0}}\in \left\{ \begin{array}{lll} \{2\}, \ i=47,50,54,55\\ \{1,2\},\ \hbox {otherwise}\end{array}\right. , \ \eta _{i}^{t_{h}}\in \left\{ \begin{array}{lll} \{2\}, \ i=47,54,55\\ \{1,2\},\ \hbox {otherwise}\end{array}\right. , ~~h\ge 1. \end{aligned}$$

### *Remark 3*

According to Example 3, the convergence speed of the proposed control algorithm can be controlled by (17). Using the traditional control^15^, it needs two state feedback controllers to make all states reach $\mathscr {E}_{e}$. However, using the nonuniform sampled-data control, it only need one state feedback controller to make all states reach $\mathscr {E}_{e}$. Therefore, it reduces the frequency of controller updates, and the amount of calculation will be reduced.

## Conclusions

In this paper, we have analyzed the nonuniform sampled-data set stabilization of DBCNs with state inequality constraints via time-variant state feedback NSDC. We have presented an effective criterion for the nonuniform sampled-data inequality constrained set reachability of DBCNs under NSDC by constructing an inequality constrained controllability matrix. By virtue of the inequality constrained reachable set and the largest inequality constrained invariant subset, we have proposed a procedure to design time-variant state feedback nonuniform sampled-data stabilizers for DBCNs. In future works, we will further investigate the stabilization and synchronization of stochastic Boolean networks with state inequality constraints by establishing a new algebraic representation. It is worth pointing out that stochastic Boolean networks with state inequality constraints have more possibilities in the state transition process, which will bring greater challenges to research.

## Data Availability

Data is provided within the manuscript or supplementary information files.

## References

[1] Wang, B., Feng, J., Li, H. & Yu, Y. On detectability of Boolean control networks. *Nonlinear Anal. Hybrid Syst.* **36**, 100859 (2020).

[2] Zhong, J., Lu, J., Liu, Y. & Cao, J. Synchronization in an array of output-coupled Boolean networks with time delay. *IEEE Trans. Neural Netw. Learn. Syst.* **25**(12), 2288–2294 (2014). 25420249 10.1109/TNNLS.2014.2305722

[3] Kauffman, S. Metabolic stability and epigenesis in randomly constructed genetic nets. *J. Theor. Biol.* **22**(3), e437 (1969). 10.1016/0022-5193(69)90015-0 5803332

[4] Zhu, Q., Gao, Z., Liu, Y. & Gui, W. Categorization problem on controllability of Boolean control networks. *IEEE Trans. Autom. Control* **66**(5), 2297–2303 (2021).

[5] Kong, X., Wang, S., Li, H. & Alsaadi, F. E. New developments in control design techniques of logical control networks. *Front. Inf. Technol. Electron. Eng.* **21**(2), 220–233 (2020).

[6] Li, B. & Lu, J. Boolean-network-based approach for the construction of filter generators. *Sci. China Inf. Sci.* **63**, 1–13 (2020).

[7] Lu, J., Li, H., Liu, Y. & Li, F. Survey on semi-tensor product method with its applications in logical networks and other finite-valued systems. *IET Control Theory Appl.* **11**(13), 2040–2047 (2017).

[8] Cheng, D., Qi, H. & Li, Z. *Analysis and Control of Boolean Networks: A Semi-Tensor Product Approach* (Springer, 2011).

[9] Zou, Y. & Zhu, J. System decomposition with respect to inputs for Boolean control networks. *Automatica* **50**(4), 1304–1309 (2014).

[10] Kong, X. & Li, H. Disturbance decoupling controller design of switched Boolean control networks in recursion. *Nonlinear Anal. Hybrid Syst.* **56**, 101558 (2025).

[11] Li, M., Lu, J., Lou, J. & Alssadi, F. The equivalence issue of two kinds of controllers in Boolean control networks. *Appl. Math. Comput.* **321**, 633–640 (2017).

[12] Li, F., Sun, J. & Wu, Q. Observability of Boolean control networks with state time delays. *IEEE Trans. Neural Netw.* **22**(6), 948–954 (2011). 21518658 10.1109/TNN.2011.2126594

[13] Zhang, L. & Zhang, K. Controllability and observability of Boolean control networks with time-variant delays in states. *Sci. China Inf. Sci.* **24**(9), 1478–1484 (2013). 10.1109/TNNLS.2013.2246187 24808585

[14] Guo, Y., Wang, P., Gui, W. & Yang, C. Set stability and set stabilization of Boolean control networks based on invariant subsets. *Automatica* **61**, 106–112 (2015).

[15] Li, R., Yang, M. & Chu, T. State feedback stabilization for Boolean control networks. *IEEE Trans. Autom. Control* **58**(7), 1853–1857 (2013).

[16] Li, H. & Pang, X. Stability analysis of large-scale Boolean networks via compositional method. *Automatica* **159**, 111397 (2024).

[17] Honda, Y., Ding, X., Mussano, F., Wiberg, A. & Nishimura, I. Guiding the osteogenic fate of mouse and human mesenchymal stem cells through feedback system control. *Sci. Rep.* **3**, 3420 (2013). 24305548 10.1038/srep03420 PMC3851880

[18] Tong, L., Liang, J. & Liu, Y. Critical observability of partially observed discrete event systems under cyber attacks. *Sci. China Inf. Sci.* **67**(2), 129203 (2024).

[19] Liu, Y., Cao, J., Sun, L. & Lu, J. Sampled-data state feedback stabilization of Boolean control networks. *Neural Comput.* **28**(4), 778–799 (2016). 26890349 10.1162/NECO_a_00819

[20] Wu, Y., Su, H., Shi, P., Shu, Z. & Wu, Z. Consensus of multiagent systems using aperiodic sampled-data control. *IEEE Trans. Cybern.* **46**(9), 2132–2143 (2016). 26316291 10.1109/TCYB.2015.2466115

[21] Zhang, X., Han, Q., Ge, X., Ning, B. & Zhang, B. Sampled-data control systems with non-uniform sampling: a survey of methods and trends. *Annu. Rev. Control* **55**, 70–91 (2023).

[22] Zhang, X., Han, Q., Zhang, B., Ge, X. & Zhang, D. Accumulated-state-error-based event-triggered sampling scheme and its application to H control of sampled-data systems. *Sci. China Inf. Sci.* **67**(6), 324–337 (2024).

[23] Tong, L., Liu, Y., Alsaadi, F. E. & Hayat, T. Robust sampled-data control invariance for Boolean control networks. *J. Franklin Inst.* **354**(15), 7077–7087 (2017).

[24] Yu, Y., Feng, J., Wang, B. & Wang, P. Sampled-data controllability and stabilizability of Boolean control networks: nonuniform sampling. *J. Franklin Inst.* **355**(12), 5324–5335 (2018).

[25] Wu, Y., Karimi, H. & Lu, R. Sampled-data control of network systems in industrial manufacturing. *IEEE Trans. Industr. Electron.* **65**(11), 9016–9024 (2018).

[26] Wu, F. Delay-independent stability of genetic regulatory networks. *IEEE Trans. Neural Netw.* **22**(11), 1685–1693 (2011). 21900072 10.1109/TNN.2011.2165556

[27] Goodwin, B. C. *Temporal Organization in Cells: A Dynamic Theory of Cellular Control Processes* (Academic Press, 1963).

[28] Han, M., Liu, Y. & Tu, Y. Controllability of Boolean control networks with time delays both in states and inputs. *Neurocomputing* **129**, 467–475 (2014).

[29] Tong, L. & Liang, J. Fault detectability of asynchronous delayed Boolean control networks with sampled-data control. *IEEE Trans. Netw. Sci. Eng.* **11**(1), 724–735 (2023).

[30] Li, Z., Chang, X. & Park, J. Quantized static output feedback fuzzy tracking control for discrete-time nonlinear networked systems with asynchronous event-triggered constraints. *IEEE Trans. Syst. Man Cybern. Syst.* **51**(6), 3820–3831 (2021).

[31] Kallak, T. K. et al. Differential gene expression in two consecutive pregnancies between same sex siblings and implications on maternal constraint. *Sci. Rep.* **14**(1), 4210 (2024). 38378837 10.1038/s41598-024-54724-3 PMC10879170

[32] Li, H. & Wang, Y. Controllability analysis and control design for switched Boolean networks with state and input constraints. *SIAM J. Control. Optim.* **53**(5), 2955–2979 (2015).

[33] Zhao, Y. & Bryson, A. State inequality constraint in the design of open and closed loop control systems. In *Navigation and Control Conference* 453–457 (1991).

[34] Palanki, S., Kravaris, C. & Wang, H. Y. Optimal feedback control of batch reactors with a state inequality constraint and free terminal time. *Chem. Eng. Sci.* **49**(1), 85–97 (1994).

[35] Wang, S. & Li, H. Column stacking approach to resolution of systems of fuzzy relational inequalities. *J. Franklin Inst.* **356**(6), 3314–3332 (2019).

[36] Chaves, M. Methods for qualitative analysis of genetic networks. In *Proceedings of the 10th European Control Conference* 671–676 (2009).

[37] Li, Y., Li, H. & Wang, S. Constrained sampled-data reachability and stabilization of logical control networks. *IEEE Trans. Circ. Syst. II Express Briefs* **66**(12), 2002–2006 (2019).

